# Notes and News

**Published:** 1902-11-15

**Authors:** 


### Notes and News.

In the King's Birthday List of Honours occurs the name of Frederick William Hewett, M.D., Anæsthetist to his Majesty, upon whom membership of the Victorian Order has been conferred.

The compulsory notification of chicken-pox within the Metropolitan area has been extended for a further period of two months, dating from November 7th. This circumstance, however, need not be taken to mean that the sanitary authorities anticipate a renewal of the small-pox epidemic. There is considerable ground for the hope that London will escape a repetition of last winter's outbreak.

Professor William Rose, F.R.C.S., has resigned his appointments as Professor of Clinical Surgery and Senior Surgeon to King's College Hospital, and has been made Member of Council and Emeritus Professor of Surgery at King's College and Consulting Surgeon to the Hospital. Mr. Watson Cheyne succeeds to the professorship of Clinical Surgery, and Mr. Albert Carless becomes Professor of Surgery and Surgeon with charge of in-patients to the Hospital.

A difference of opinion has arisen at Dundee between the Works Committee of the Town Council and the directors of the Royal Infirmary with regard to a proposed extension of the Infirmary buildings for the reception of cancer cases. Mr. J. K. Caird had offered to defray the cost of the new wards, plans being prepared at his request, over which difficulties have arisen. At a recent conference between the Works Committee and the directors a resolution disapproving of the plans was carried by the casting vote of the chairman.

It is proposed that a memorial to those students of Guy's Hospital who lost their lives during the South African War shall be placed in the Hospital colonnade. The memorial will be in the form of a drinking fountain, designed by Mr. F. Wheeler, F.R.I.B.A. The list at present is as follows:—H. C. Bryant, H. Bernard Onraet, F. T. FitzHugh, L. J. Hughes, T. Jones, H. Pope-Walker, F. M. Russell, C. B. Sells, F. Welford, and S. Whicher. The Committee are anxious to know as soon as possible of any names which should be added to these.

Dr. Lipponi, medical adviser to the Pope, has been successfully operated upon for appendicitis by Professor Mazzoni.

The Governor of Mauritius, Sir Charles Bruce, reports for the week ending November 6th, 28 cases of plague, of which 14 were fatal.

The *Contemporary Review* for November contains a comprehensive reply by Mr. Stephen Paget to the "Open Letter to the Registrar-General" of Mr. Stephen Coleridge in the October issue.

A new cottage hospital and dispensary was recently opened at Abergavenny by Lord Tredegar. The building is to be known as the "Victoria Hospital," in memory of the late Queen.

Dr. E. F. Bashford, assistant to Professor Sir T. R. Fraser, of Edinburgh, has been appointed by the executive committee of the Cancer Research Fund, from amongst a number of applicants, to the post of Superintendent of Cancer Research.

The next general meeting of the Medico-Psychological Association of Great Britain and Ireland takes place on Thursday, November 20th, at 11 Chandos Street, at 3 p.m. After the meeting Sir William Gowers will give an address on "Sanity and Insanity, Lunacy and Law," whilst other papers will be contributed by Dr. W. Lloyd Andriezen and Dr. Edwin Goodall.

It is a matter for much congratulation that the Metropolitan sanitary authorities are being urged by the London County Council to inspect and examine the kitchens at eating-houses and restaurants, and that these bodies are taking up this important piece of work with some vigour. Dr. Collingridge, Medical Officer of the City of London, deals with this subject in his last report, giving statistics which show that inspection is urgently needed. The thousands of Londoners who daily make use of the public restaurants will eat their meals with greater relish for the knowledge that the regions where their food is prepared are under proper supervision.

The campaign against the spread of tuberculosis is being carried on with vigour in all parts of the country, and there are many plans on foot for the further establishment of sanatoria for the treatment of the disease. A sanatorium for the reception of 50 patients is to be built in Northumberland at a cost of £50,000; the foundation stone has recently been laid of a similar institution at Delamere, the expenses of erection to be borne by Mr. W. T. Crossley, on condition of public endowment; while yet a third scheme provides for the needs of Gloucestershire, Somerset, and Wilts, the site for the proposed building being on the Wiltshire Downs, near Winsley. Signs also are not wanting to show that the public generally are more fully alive than heretofore to the dangers of the disgusting habit of spitting, so freely indulged in by a section of the community. A witness was recently sent out of court for expectorating, Judge Emden ordering the prompt application of disinfectants, and remarking that such a proceeding in public places was not only filthy but dangerous.

